# Social Determinants of Health and Clinical Outcomes in Hypertrophic Cardiomyopathy

**DOI:** 10.1001/jamacardio.2025.4869

**Published:** 2026-01-07

**Authors:** Neha Hafeez, Brian L. Claggett, Anjali T. Owens, Adam S. Helms, Sara Saberi, Rachel Lampert, John C. Stendahl, Euan A. Ashley, Victoria N. Parikh, Neal K. Lakdawala, Jodie Ingles, Iacopo Olivotto, Carolyn Y. Ho, Matthew R. Taylor, Sadiya S. Khan, Sharlene M. Day

**Affiliations:** 1 Division of Cardiology, Department of Internal Medicine, University of Pennsylvania, Philadelphia; 2 Department of Medicine, Brigham and Women’s Hospital, Boston, Massachusetts; 3 Division of Cardiovascular Medicine, Department of Internal Medicine, University of Michigan, Ann Arbor; 4 Section of Cardiovascular Medicine, Department of Medicine, Yale School of Medicine, New Haven, Connecticut; 5 Center for Inherited Cardiovascular Disease, Division of Cardiovascular Medicine, Stanford University School of Medicine, Stanford, California; 6 Centre for Population Genomics, Garvan Institute of Medical Research and University of New South Wales, Sydney, New South Wales, Australia; 7 Meyer’s Children’s Hospital, IRCCS, University of Florence, Florence, Italy; 8 Adult Medical Genetics Program, Division of Cardiology, University of Colorado Anschutz Medical Campus, Aurora; 9 Departments of Medicine and Preventive Medicine, Feinberg School of Medicine, Northwestern University, Chicago, Illinois; 10 Associate Editor, *JAMA Cardiology*; 11 Associate Editor for Translational Science, *JAMA Cardiology*

## Abstract

**Importance:**

Area-based indicators of social determinants of health (SDOH) are associated with higher risk for acquired heart disease, but their impact on conditions with a strong genetic etiology, such as hypertrophic cardiomyopathy (HCM), is not well understood.

**Objective:**

To determine the association of area-based SDOH with clinical outcomes in patients with HCM.

**Design, Setting, and Participants:**

This multicenter, prospective cohort study was conducted among US adult patients with HCM from 5 sites in the Sarcomeric Human Cardiomyopathy Registry (a multicenter prospective registry of patients with HCM) who were followed up for a median (IQR) period of 2.15 (0.15-5.82) years. Data were entered from 2015 to March 2024, and data analysis was completed from March 2024 to June 2025.

**Exposures:**

Patients’ residential addresses were geocoded at the zip code level and linked to the American Communities Survey to estimate area-based (1) median household income and (2) social deprivation index (SDI), which ranges from 0 to 100, with higher scores indicating a more deprived area.

**Main Outcomes and Measures:**

Multivariate models, adjusting for age at diagnosis, body mass index, hypertension, and sex, were used to estimate the independent association of area-based median household income and SDI with heart failure (HF), ventricular arrhythmias (VA), and an overall composite outcome (VA, HF, atrial fibrillation, stroke, and death).

**Results:**

Among 4431 US adult patients with HCM, median (IQR) age at HCM diagnosis was 51.3 (38.9-61.6) years, and 1862 patients (42.0%) were female. Median (IQR) area-based household income was $80 000 ($60 000-$110 000), and median (IQR) SDI was 25 (10-55). Adjusted hazard ratios comparing the lowest income group to the highest income group were 2.07 (95% CI, 1.77-2.42; *P* < .001) for HF, 1.31 (95% CI, 0.97-1.78; *P* = .08) for VA, and 1.52 (95% CI, 1.36-1.69; *P* < .001) for the overall composite outcome. Adjusted hazard ratios comparing the highest SDI (ie, more deprived) group to the lowest SDI group were 1.48 (95% CI, 1.29-1.70; *P* < .001) for HF, 1.55 (95% CI, 1.15-2.09; *P* = .004) for VA, and 1.36 (95% CI, 1.22-1.50; *P* < .001) for the overall composite outcome.

**Conclusions and Relevance:**

In this multicenter cohort study, residing in an area with lower median household income or worse SDI were each independently associated with adverse clinical outcomes in patients with HCM. These findings suggest that despite the genetically determined nature of HCM, place of residence is associated with patient outcomes.

## Key Points

**Question:**

Are area-based social determinants of health (SDOH) associated with cardiovascular outcomes in patients with hypertrophic cardiomyopathy (HCM)?

**Findings:**

In this cohort study that included 4431 adult patients with HCM, residing in an area with either lower median household income or worse social deprivation index was significantly associated with adverse cardiovascular outcomes (heart failure, ventricular and atrial arrhythmias, stroke, and death).

**Meaning:**

Area-based SDOH may be useful for risk assessment in patients with HCM and may inform strategies to reduce health disparities and improve outcomes.

## Teaser

This cohort study using data from the Sarcomeric Human Cardiomyopathy Registry determines the association of area-based social determinants of health with clinical outcomes in patients with hypertrophic cardiomyopathy.

## Introduction

Social determinants of health (SDOH) encompass a broad array of individual- and neighborhood-level factors, including income, education, and housing, which together impact an individual’s risk for developing cardiovascular diseases and subsequent health outcomes.^1,2,3,4^ Patients with adverse SDOH often face greater challenges in accessing high-quality health care, including preventive services, diagnostic testing, and timely interventions.^5^ Adverse SDOH are associated with many cardiovascular risk factors, including obesity, hypertension, and diabetes.^6,7,8^ In addition, emerging evidence suggests that allostatic load,^9^ or the cumulative physiologic toll of the stress of deprivation defined by biologic markers, such as autonomic dysregulation secondary to excess stress hormones,^10,11^ immune cell function, and cellular aging, may mediate the associations between SDOH and risk of acquired cardiovascular disease.^12,13^

While prior studies have investigated the role of SDOH in influencing outcomes in acquired cardiovascular disease, it remains unclear if the influence of SDOH on cardiovascular outcomes would extend to conditions with a strong inherited etiology, such as hypertrophic cardiomyopathy (HCM). HCM is defined by left ventricular (LV) hypertrophy, diastolic dysfunction, and myocardial fibrosis.^14^ Clinical presentation is highly variable, where some individuals are completely asymptomatic, while others experience heart failure and/or life-threatening arrhythmias.^15,16^ This heterogeneity in disease expression is only partially explained by clinical factors, such as age at diagnosis, sex, obesity, and genetic status, and may imply a role for environmental or social factors.^17,18,19,20,21,22^ The objective of this study was to determine if area-based SDOH are independently associated with clinical outcomes in adult patients with HCM.

## Methods

### Sarcomeric Human Cardiomyopathy Registry and Participating Sites

The Sarcomeric Human Cardiomyopathy Registry (SHaRe), a prospective multisite international registry containing clinical and genetic information on patients with HCM, was leveraged for this study. SHaRe is made up of 14 adult and pediatric HCM centers in 6 countries (US, Italy, the Netherlands, UK, Denmark, and Australia), with more than 12 000 patients with HCM and more than 80 000 patient-years of follow-up.^23,24^ Given the focus on US-based zip code–level exposures for this analysis, patient records from all adult sites in the US (University of Pennsylvania, University of Michigan, Stanford University, Yale University, and Brigham and Women’s Hospital) were analyzed, with data last updated in March 2024. Deidentified data were collected either under a waiver of consent or after informed consent was provided, depending on site-specific institutional review board requirements. The study conforms to the principles of the Declaration of Helsinki and was approved by each local institutional review board.^23,24^

### Study Population

Inclusion criteria for patients were defined as (1) site-designated diagnosis of HCM defined by the presence of LV wall thickness of 15 mm or more (13 mm if genotype positive) in the absence of abnormal loading conditions, (2) aged 18 years or older, (3) availability of residential zip codes for geocoding area-based SDOH metrics, and (4) presence of at least 2 SHaRe site encounters. Clinical records included sex, age at diagnosis, New York Heart Association (NYHA) class, body mass index (BMI, calculated as weight in kilograms divided by height in meters squared), comorbidities, family history, genetics, echocardiography, and clinical events (Table). Among the 4520 adult patients with HCM enrolled in SHaRe US sites, 4431 met the inclusion criteria (98.0%), and 89 patients were excluded due to missing zip codes. Sarcomere-positive variant status was defined as those with genetic testing demonstrating a pathogenic or likely pathogenic variant in 1 of 9 contractile protein genes (*MYH7*, *MYBPC3*, *TNNT2*, *TNNI3*, *TPM1*, *MYL2*, *MYL3*, *ACTC1*, or *TNNC1*). Patients with variants of unknown significance and pathogenic variants in nonsarcomere genes (including genes associated with metabolic disease, storage disease, or syndromic causes of LV hypertrophy) were not included in this analysis.

**Table.** Baseline Characteristics^a^

Characteristic	No. (%)				*P* value
	Overall (N = 4431)	Income category			
		Lowest income (<$80 000) (n = 1763)	Middle group ($80 000-$100 000) (n = 1412)	Highest income (>$100 000) (n = 1256)	
Age at diagnosis, mean (SD), y	50.1 (15.3)	49.2 (15.2)	50.6 (15.3)	50.7 (15.5)	.009
BMI >25^b^	3601 (81.3)	1500 (85.1)	1145 (81.1)	956 (76.1)	<.001
Sex					
Female	1862 (42.0)	798 (45.3)	593 (42.0)	471 (37.5)	<.001
Male	2569 (58.0)	965 (54.7)	819 (58.0)	785 (62.5)	
Race^c^					
African American or Black	383 (8.6)	270 (15.3)	81 (5.7)	32 (2.6)	<.001
Asian	221 (5.0)	34 (1.9)	62 (4.4)	125 (10.0)	<.001
White	3420 (77.2)	1328 (75.3)	1127 (79.8)	965 (76.8)	.01
NYHA class III or IV symptoms	1157 (26.1)	594 (33.7)	349 (24.7)	214 (17.0)	<.001
Hypertension	2363 (53.3)	992 (56.3)	753 (53.3)	618 (49.2)	<.001
Genetic testing	2702 (61.0)	1050 (59.6)	877 (62.1)	775 (61.7)	.28
P/LP variant (% of tested)	1080 (40.0)	449 (42.8)	356 (40.6)	275 (35.5)	.006
VUS (% of tested)	529 (19.6)	201 (19.1)	167 (19.0)	161 (20.8)	.61
SCD family history	218 (4.9)	78 (4.4)	62 (4.4)	78 (6.2)	.05
Echocardiography, mean (SD)					
LAD, mm	42.7 (10.2)	44.3 (9.7)	41.8 (10.1)	41.1 (10.8)	<.001
Maximum LVWT, mm	16.6 (4.5)	17.2 (4.7)	16.5 (4.3)	15.7 (4.2)	<.001
LVEF, %	64.8 (9.5)	65.1 (9.6)	64.5 (9.6)	64.6 (9.0)	.13
LVOT gradient, mm Hg	42.0 (43.5)	44.7 (44.7)	41.8 (43.9)	37.9 (40.5)	.001
**Characteristic**	**No. (%)**				***P* value**
	**Overall (N = 4431)**	**SDI category**			
		**Most deprived (SDI >45) (n = 1385)**	**Middle group (SDI 20-45) (n = 1382)**	**Least deprived (SDI <20) (n = 1664)**	
Age at diagnosis, mean (SD), y	50.1 (15.3)	49.2 (15.4)	50.1 (15.6)	50.8 (15.0)	.01
BMI >25^b^	3601 (81.3)	1158 (83.6)	1131 (81.8)	1312 (78.8)	.003
Sex					
Female	1862 (42.0)	606 (43.8)	590 (42.7)	666 (40.0)	.10
Male	2569 (58.0)	779 (56.2)	792 (57.3)	998 (60.0)	
Race^c^					
African American or Black	383 (8.6)	275 (19.9)	68 (4.9)	40 (2.4)	<.001
Asian	221 (5.0)	46 (3.3)	82 (5.9)	93 (5.6)	.002
White	3420 (77.2)	912 (65.9)	1093 (79.1)	1415 (85.0)	<.001
NYHA class III or IV symptoms	1157 (26.1)	422 (30.5)	351 (25.4)	384 (23.1)	<.001
Hypertension	2363 (53.3)	769 (55.5)	759 (54.9)	835 (50.2)	.005
Genetic testing	2702 (61.0)	780 (56.3)	855 (61.9)	1067 (64.1)	<.001
P/LP variant (% of tested)	1080 (40.0)	312 (40.0)	358 (41.9)	410 (38.4)	.31
VUS (% of tested)	529 (19.6)	183 (23.5)	152 (17.8)	194 (18.2)	.006
SCD family history	218 (4.9)	73 (5.3)	78 (5.6)	67 (4.0)	.09
Echocardiography, mean (SD)					
LAD, mm	42.7 (10.2)	43.0 (10.6)	42.1 (10.5)	42.9 (9.6)	.06
Maximum LVWT, mm	16.6 (4.5)	17.1 (4.7)	16.4 (4.5)	16.3 (4.3)	<.001
LVEF, %	64.8 (9.5)	65.3 (9.3)	64.4 (9.6)	64.7 (9.5)	.06
LVOT gradient, mm Hg	42.0 (43.5)	43.1 (43.5)	44.3 (44.2)	39.2 (42.8)	.01

Abbreviations: BMI, body mass index; LAD, left atrial diameter; LVEF, left ventricular ejection fraction; LVOT, left ventricular outflow tract; LVWT, left ventricular wall thickness; NYHA, New York Heart Association; P/LP, pathogenic or likely pathogenic; SCD, sudden cardiac death; VUS, variants of unknown significance.

^a^ Characteristics obtained from first visit documented in the Sarcomeric Human Cardiomyopathy Registry. Top panel represents characteristics in patients divided by income, and bottom panel represents characteristics in patients divided by social deprivation index.

^b^ Calculated as weight in kilograms divided by height in meters squared.

^c^ Self-reported by participants. Other categories not listed due to small sample sizes include American Indian or Alaskan Native, Native Hawaiian or Other Pacific Islander, and Other.

### Geocoded Median Household Income and Social Deprivation Index

Two key metrics of SDOH were selected and defined at the zip code level for each patient: median household income and social deprivation index (SDI). Patients were divided into groups determined by tertiles of area-based metrics of SDOH.^25^ SDI is based on the following 7 components: percentage living in poverty, percentage with fewer than 12 years of education, percentage of single-parent households, percentage living in rented housing units, percentage living in an overcrowded housing unit, percentage of households without a car, and percentage of nonemployed adults under 65 years of age.^26,27^ SDI by zip code was obtained from the Robert Graham Center (based on the most recent American Community Survey’s 5-year estimates, published in 2019). The SDI value assigned ranged from 0 to 100, with higher values reflecting greater deprivation.^28,29^ SDI was specifically selected as the composite index of interest because this was recently used in the development of the PREVENT equations, which are intended to predict risk for acquired cardiovascular disease.^30,31^ Median household income by zip code was obtained from a commercial vendor (US Census Bureau data 2023, from Cubit Planning).^32^ Notably, both databases convert zip codes to zip code tabulation areas (ZCTAs) to determine median household income and SDI in that area. Some zip codes were missing from both databases due to the following issues: (1) zip code listed was for a PO box or business-only area or (2) the zip code was new, and the US Census Bureau had not yet created a corresponding ZCTA. Patients without a valid ZCTA code based on their abstracted zip code were excluded from the study (n = 89).

### Definition of Study Outcomes

As previously identified and published in other studies using the SHaRe database, the following 4 primary composite outcomes were defined: (1) heart failure (HF) composite: first occurrence of NYHA class III or IV symptoms, cardiac transplant, or LV assist device implant; (2) ventricular arrhythmia (VA) composite: first occurrence of sudden cardiac death, resuscitated cardiac arrest, or appropriate implantable cardioverter-defibrillator therapy; (3) atrial fibrillation (AF) composite; and (4) overall composite: first occurrence of any component of the HF, VA, and AF composites, all-cause mortality, and stroke.^19,22,23^ Hazard ratios (HRs) for the occurrence of clinical outcomes were adjusted for age at diagnosis, BMI, hypertension, and sex.

### Statistical Analysis

All time-to-event analyses were performed from the time participants entered the SHaRe dataset (or age 18 years if entered prior to adulthood) to the time of first occurrence of any component of each outcome as previously described. Cox multivariate analysis was performed using all candidate variables. A 2-sided *P* value less than .05 was considered statistically significant. All analyses were performed using Stata version 18.0 software (StataCorp).

## Results

### Study Population

Among the 4431 included patients from 5 US sites, median (IQR) age at HCM diagnosis was 51.3 (38.9-61.6) years, and 1862 participants (42.0%) were female. Median (IQR) follow-up duration was 2.15 (0.15-5.82) years (Table). Median (IQR) area-based household income was $80 000 ($60 000-$110 000), and median (IQR) SDI was 25 (10-55). Patients were divided into tertiles by area-based income, with the lowest income group defined as residing in an area with household income less than $80 000, the middle group defined as income of $80 000 to $100 000, and the highest income group defined as income greater than $100 000. Compared to those in the highest income group, patients residing in the lowest income group had a younger mean (SD) age at diagnosis (49.2 [15.2] years vs 50.7 [15.5] years; *P* = .009), a higher percentage of overweight or obesity (BMI ≥25; 85.1% vs 76.1%; *P* < .001), and a higher percentage of NYHA class III or IV symptoms (33.7% vs 17.0%; *P* < .001) (Table). Patients were divided into tertiles by SDI, with the most deprived group defined as areas with SDI greater than 45, the middle group defined as SDI of 20 to 45, and the least deprived group defined as SDI of less than 20. Compared to those in the least deprived group, patients in the most deprived areas had younger mean (SD) age at HCM diagnosis (49.2 [15.4] years vs 50.8 [15.0] years; *P* = .01), a higher percentage with overweight or obesity (83.6% vs 78.8%; *P* < .001), and a higher percentage with NYHA class III or IV symptoms (30.5% vs 23.0%; *P* < .001) (Table). Patients who self-identified as Black (8.6% of the study population) were more likely to reside in a lower income area (15.3% of lowest income area vs 2.6% of the highest income area; *P* < .001) and were more likely to reside in a higher SDI area (19.9% of the most deprived area vs 2.4% of the least deprived area; *P* < .001) compared to patients who identified as Asian or White (Table).

Patients residing in a lower income area compared to a higher income area had a greater mean (SD) left atrial diameter (44.3 [9.7] mm in lowest income area vs 41.1 [10.8] mm in highest income area; *P* < .001), greater mean (SD) LV wall thickness (17.2 [4.7] mm vs 15.7 [4.2] mm; *P* < .001), and higher mean (SD) LV outflow tract (LVOT) gradient (44.7 [44.7] mm Hg vs 37.9 [40.5] mm Hg; *P* = .001). Patients residing in a higher SDI area had a greater mean (SD) LV wall thickness (17.1 [4.7] mm in the most deprived area vs 16.3 [4.3] mm in the least deprived area; *P* < .001) and higher LVOT gradient (43.1 [43.5] mm Hg vs 39.2 [42.8] mm Hg; *P* = .01) (Table).

### Median Household Income and Outcomes

**Figure 1.**
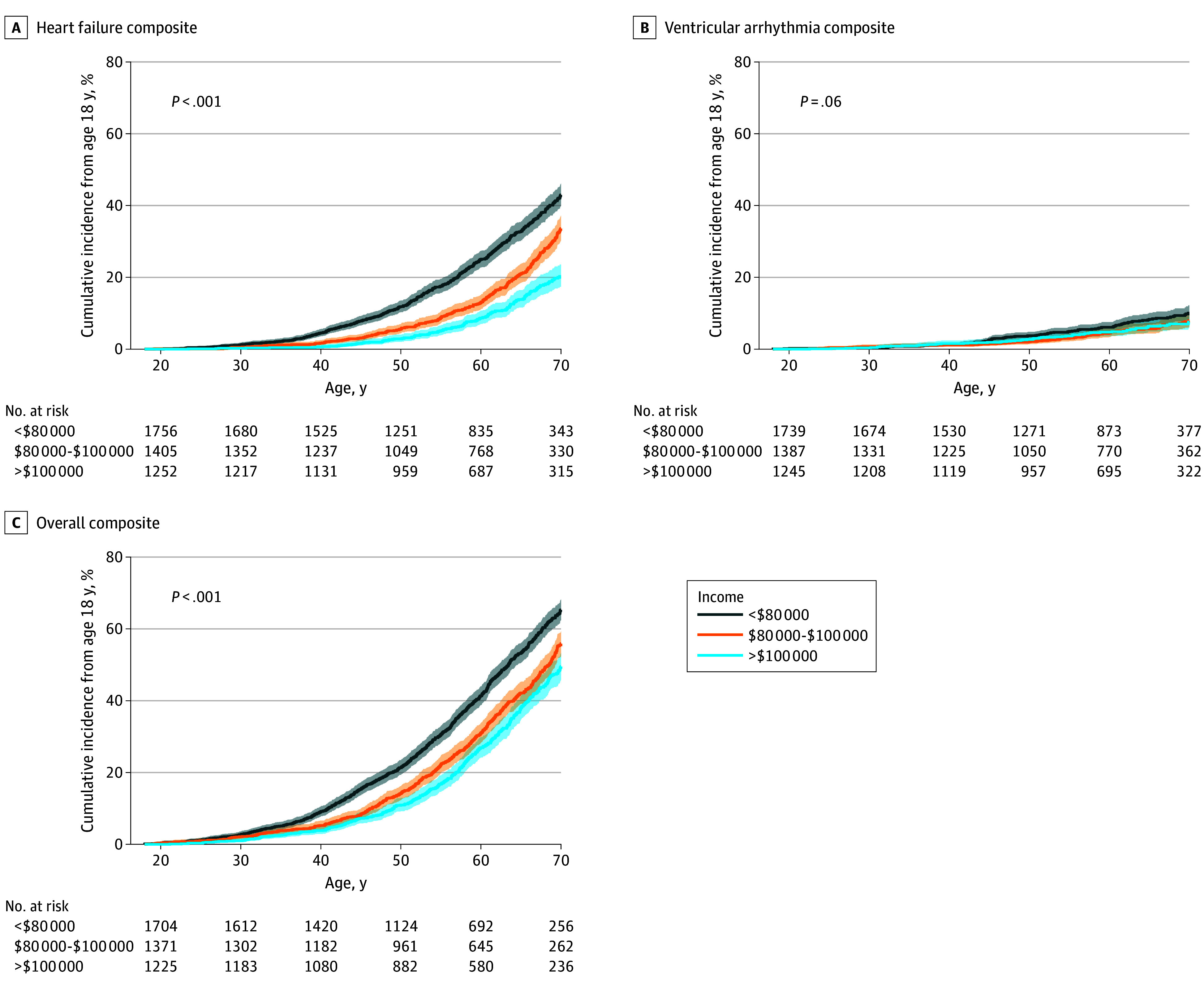
Association of Household Median Income and Clinical Outcomes in Patients With Hypertrophic Cardiomyopathy Cumulative incidence of events from age 18 years for outcomes of interest (heart failure composite [A], ventricular arrhythmia composite [B], and overall composite [C]), stratified by household median income <$80 000, $80 000-$100 000, and >$100 000. Shaded areas indicate 95% confidence intervals. Statistical significance was determined by multivariate Cox regression models correcting for age, sex, hypertension, and obesity.

For the HF composite outcome, HRs were 2.07 (95% CI, 1.77-2.42; *P* < .001) and 1.42 (95% CI, 1.24-1.62; *P* < .001) for patients residing in the lowest income areas compared to the highest and middle income areas, respectively (Figure 1A). For the VA composite outcome, HRs were 1.31 (95% CI, 0.97-1.78; *P* = .08) and 1.28 (95% CI, 0.95-1.73; *P* = .09) for patients residing in the lowest income areas compared to the highest and middle income areas, respectively (Figure 1B). For AF, HRs were 1.10 (95% CI, 0.97-1.26; *P* = .14) and 1.19 (95% CI, 1.04-1.37; *P* = .01) for patients living in the lowest income area compared to the highest and middle income areas, respectively (eFigure 1A in Supplement 1). For the overall composite outcome, HRs were 1.52 (95% CI, 1.36-1.69; *P* < .001) and 1.26 (95% CI, 1.14-1.39; *P* < .001) for patients residing in the lowest income areas compared to the highest and middle income areas, respectively (Figure 1C).

### SDI and Outcomes

**Figure 2.**
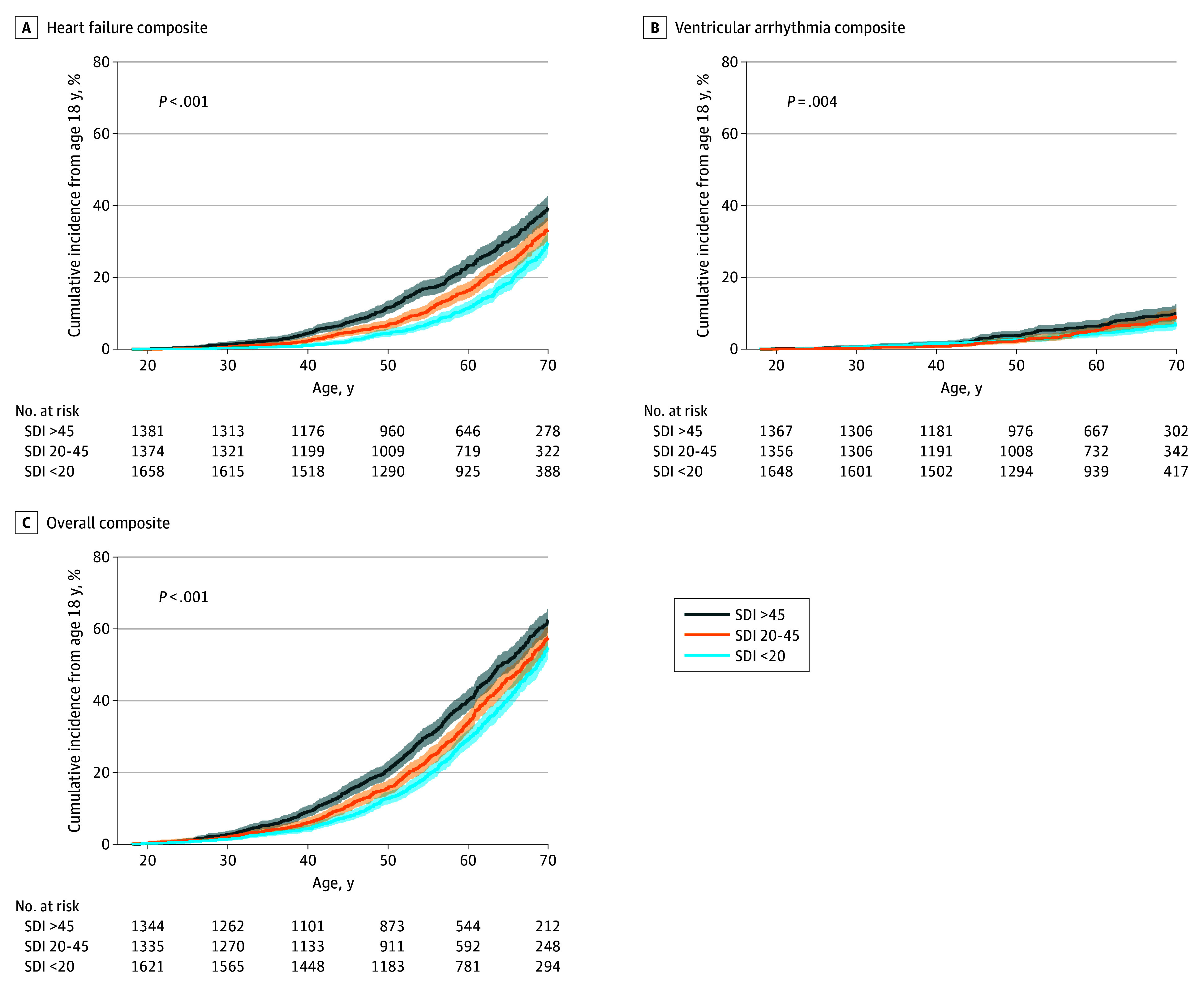
Association of Social Deprivation Index (SDI) With Clinical Outcomes in Patients With Hypertrophic Cardiomyopathy Cumulative incidence of events from age 18 years for outcomes of interest (heart failure composite [A], ventricular arrhythmia composite [B], and overall composite [C]), stratified by SDI >45, 20-45, and <20. Shaded areas indicate 95% confidence intervals. Statistical significance determined by multivariate Cox regression models correcting for age, sex, hypertension, and obesity.

For the HF composite outcome, HRs were 1.48 (95% CI, 1.29-1.70; *P* < .001) and 1.28 (95% CI, 1.12-1.48; *P* < .001) for patients residing in the most deprived areas compared to the least and middle deprived areas, respectively (Figure 2A). For the VA composite outcome, HRs were 1.55 (95% CI, 1.15-2.09; *P* = .004) and 1.17 (95% CI, 0.87-1.58; *P* = .31) for patients residing in the most deprived areas compared to the least and middle deprived areas, respectively (Figure 2B). For the AF composite, HRs were 1.16 (95% CI, 1.01-1.33; *P* = .04) and 0.88 (95% CI, 0.77-1.01; *P* = .07) for patients residing in the most deprived areas compared to the least and middle deprived areas, respectively (eFigure 1B in Supplement 1). For the overall composite outcome, HRs were 1.36 (95% CI, 1.22-1.50; *P* < .001) and 1.13 (95% CI, 1.02-1.26; *P* = .02) for patients residing in the most deprived areas compared to the least and middle deprived areas, respectively (Figure 2C).

### Sarcomere-Positive Genetic Status, SDOH, and Outcomes

**Figure 3.**
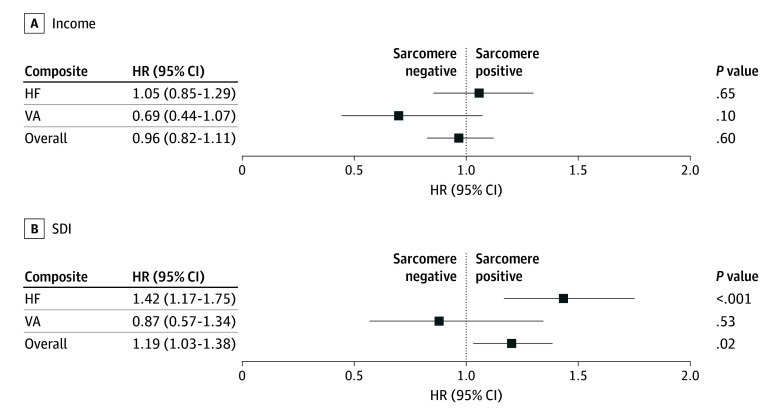
Relative Hazard of Social Deprivation Index (SDI) and Income on Clinical Outcomes, Stratified by Presence or Absence of a Pathogenic Sarcomere Variant Forest plot demonstrating hazard ratio (HR) of cumulative incidence of events from age 18 years for outcomes of interest, comparing HR of outcomes of sarcomere-positive vs sarcomere-negative patients based on income (A) or SDI (B). Error bars represent 95% confidence intervals. HF indicates heart failure; VA, ventricular arrhythmia.

Patients residing in a higher SDI area were less likely to have undergone genetic testing than patients from a lower SDI area (56.3% vs 64.1%; *P* < .001); however, there was no significant difference in the detection rate of pathogenic or likely pathogenic variants on testing by SDI. In contrast, there was no significant difference with rates of genetic testing of patients residing in a lower income area compared to a higher income area (59.6% vs 61.7%; *P* = .28), but there was a significantly higher detection rate of pathogenic or likely pathogenic variants in the lower income vs the higher income groups (42.8% vs 35.5%; *P* = .006) (Table). The interaction of sarcomere variant status with area-based income or SDI for each composite outcome after correcting for age at diagnosis, BMI, hypertension, and sex was evaluated to assess for any differential impact of genetic status in driving the association between SDOH and clinical outcomes previously mentioned. There was a significant interaction between the presence of a sarcomere variant (vs no sarcomere variant) with SDI for the HF composite (HR, 1.42; 95% CI, 1.17-1.75; *P* < .001) and for the overall composite (HR, 1.19; 95% CI, 1.03-1.38; *P* = .02) (Figure 3A). This implies that SDI had a greater influence on the HF and overall composite outcomes in patients with a sarcomere variant. This association was not seen when comparing SDI and the VA outcome (Figure 3A) or the AF outcome (eFigure 2 in Supplement 1). The association between area-based income and clinical outcomes was not significantly different by sarcomere status (Figure 3B).

### Household Income and SDI in Surrounding Areas

**Figure 4.**
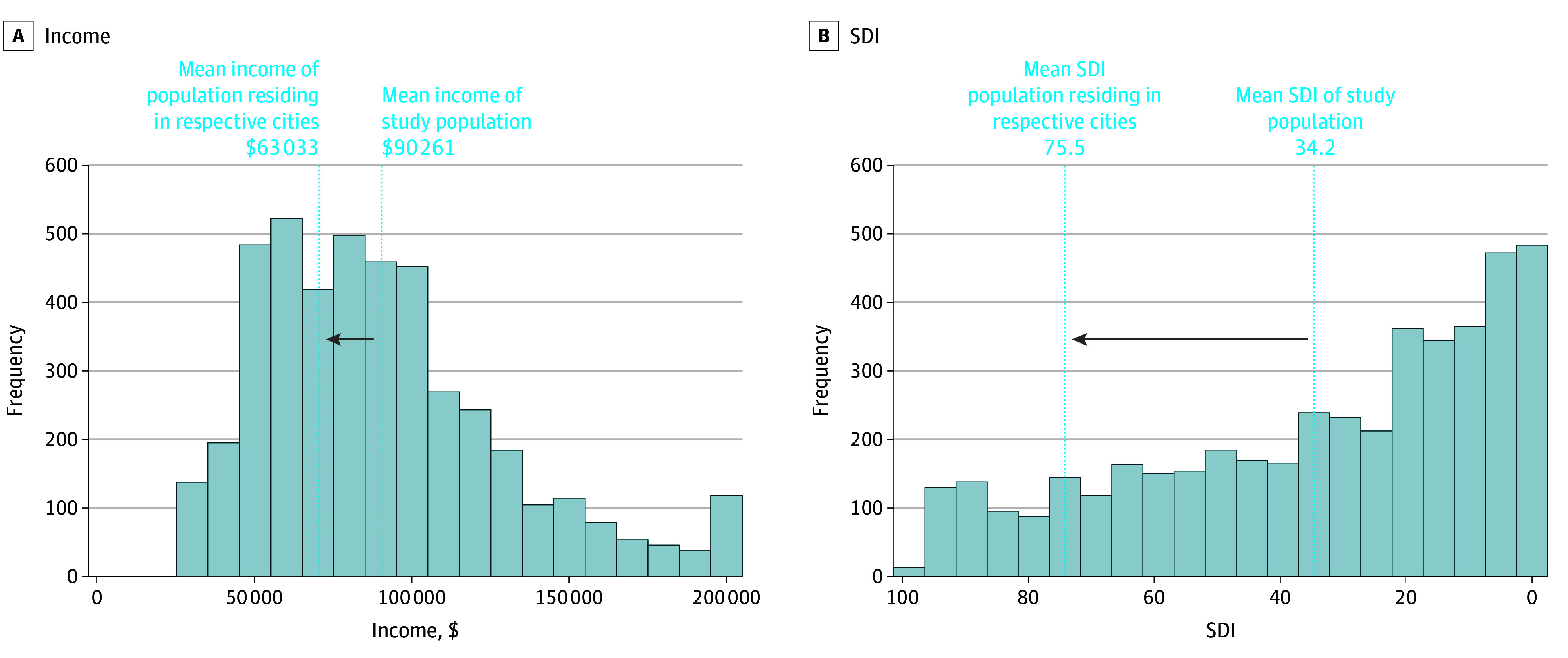
Discrepancies Between Mean Social Deprivation Index (SDI) and Income in the Sarcomeric Human Cardiomyopathy Registry Study Population Compared to the Population in the Cities in Which Each Hospital Resides A, Histogram depicting breakdown of income of sample population included in this study. The dotted line on the right represents the mean income of the sample population. The dotted line indicated by the arrow represents the mean income of the population living in the city of each respective hospital included in the study. B, Histogram depicting breakdown of SDI of sample population included in this study. The dotted line on the right represents the mean SDI of the sample population. The dotted line indicated by the arrow represents the mean SDI of the population living in the city of each respective hospital included in this study.

The mean household incomes and SDI of the cities included in the respective SHaRe sites were calculated. Mean (SD) household income in the study population was $90 261 ($38 992) compared to $63 033 in the general population of the 5 included cities (Figure 4A). The mean (SD) SDI within our study population was 34.2 (28.5) compared to 75.5 in the general population of those cities (Figure 4B). Taken together, patients living in an area with a lower income and higher SDI compared to the general population were underrepresented in SHaRe centers.

## Discussion

While the link between adverse SDOH and risk of acquired cardiovascular disease, such as atherosclerotic cardiovascular disease and HF, is well established,^33^ it was unclear whether SDOH would be associated with disease trajectory in patients with HCM, a condition driven by a strong genetic etiology. We indeed observed an independent association between area-level social factors and adverse outcomes in patients with HCM, even after adjusting for differences in age, sex, and clinical risk factors with BMI and hypertension. These findings are consistent with a 2025 study^34^ that showed an association between lower median household income and higher all-cause mortality, defibrillator shocks, and cardiac transplant in patients with HCM referred to a single specialty center. The present study extends these associations to include patients from multiple institutions across the US, incorporates genotype status, provides granular clinical outcomes data from a prospective registry with longitudinal follow-up, and includes a broader composite metric of area-based socioeconomic status (ie, SDI).

There are likely multiple factors that contribute to the higher observed adverse outcomes in patients with HCM who reside in more deprived areas. One potential factor may be limited access to expert care, which could result in delayed diagnosis and implementation of guideline-directed therapies before disease progression.^35,36^ The observation that patients residing in areas of higher social deprivation and/or lower median household incomes have earlier age at diagnosis and markers of greater disease severity implies earlier disease manifestation and more accelerated disease course in these individuals. Current guidelines support early access to expert centers for patients with HCM to optimize their care and the care of their family members.^37^ The finding that patients seen at SHaRe expert centers lived in areas with higher income and lower SDI compared to the general population in regions serviced by those hospitals further supports restricted access to care, consistent with a prior study performed at an expert HCM center in Sydney, Australia.^38^

Other potential factors that may contribute to the association between lower area-based socioeconomic status and adverse outcomes in patients with HCM include higher chronic stress levels, lack of a social support network, challenges adhering to healthy lifestyle practices, financial constraints that affect medication adherence, transportation, availability for regular medical visits, and insurance coverage. These factors are particularly important in the current era of HCM therapies. For example, cardiac myosin inhibitors are now standard of care for treating patients with symptomatic LVOT obstruction.^37^ However, cost, need for reliable insurance, and availability to attend frequent clinic visits and echocardiograms may be barriers for patients with adverse SDOH. This is especially concerning, given that patients residing in areas with lower incomes and higher SDI have significantly higher LVOT gradients compared to those from areas with higher incomes and lower SDI (Table), suggesting that the patients who need treatment more may be less likely to be able to access it.

Previous studies have shown that patients with HCM due to sarcomere variants have an earlier onset of disease and often more severe clinical phenotypes compared to those without sarcomere variants.^39,40,41,42^ In this study, we observed that residing in an area with high SDI had a greater impact on patients with sarcomere variants for the HF composite and overall composite outcomes in patients with sarcomere variants compared with those who were genotype negative. The reasons for this are unclear but may relate to greater vulnerability of sarcomeric HCM in general, which is then magnified by potentially delayed access to expert care and poor social support. As this association was not observed between sarcomere genetic status and area-based median household incomes, it is possible that other components of SDOH that are part of the SDI are driving this interaction. Alternatively, this could be explained by the somewhat limited range in income brackets compared to other components of the SDI.

We observed a higher detection rate of pathogenic or likely pathogenic sarcomeric variants in patients residing in the lower income areas compared to the higher income areas, despite similar rates of genetic testing across income groups. Patients living in areas with lower household incomes tended to have a more severe phenotype, with greater maximal wall thickness and left atrial diameter. The higher disease severity in patients residing in lower income areas may reflect a higher threshold for referral to expert centers or financial barriers in accessing routine care at these centers. The higher severity of disease may in turn explain the greater proportion of sarcomeric disease compared to patients living in higher income areas, who may have experienced a lower threshold for being seen at expert centers.

In our sample, self-reported race was associated with residing in a more deprived area, with individuals who self-identified as Black being more likely to live in a higher SDI or lower income area. This is due to the long-standing legacy of structural racism (eg, redlining) in the US resulting in racial residential segregation that persists today. By definition, race is a social construct, and there is no genetic interpretation of race; therefore, there is no biologic susceptibility of disease based solely on race.^43,44,45^ However, given that race is interconnected with SDOH, it may serve as a marker or proxy of SDOH that is associated with clinical expression, access to care, and outcomes in HCM.^46,47^ Regardless, because the goal of this study was to focus on a more direct and proximate measure of an individual’s environment and lived experience with area-based SDOH, race was a priori not included as a covariate in this study. Moreover, this approach avoids reifying biologic misinterpretations of race and underscores the need to address the modifiable drivers of inequities in clinical outcomes for acquired and genetic conditions.

### Limitations

There are some limitations to this study. First, area-based SDOH was derived at the zip code level, which represents an average area of 90 square miles, across which significant heterogeneity may still exist at the neighborhood or block level where an individual resides. Moreover, area-based SDOH metrics do not account for differences in individual-level SDOH that may also influence disease outcomes.^48^ Second, only US sites are included because of the relevance of the area-based exposures for zip code–based household income and SDI data. Additionally, there are large differences between social services available in sites outside the US and across countries. Third, the zip code was obtained from the last clinic visit and based on SDOH derived from the 5-year summary in the American Community Survey (2014-2018) and does not account for geographic or temporal changes in area-based SDOH. Fourth, we selected SDI as an area-based composite index of SDOH because of its prior use in the PREVENT equations, but many area-based indices exist (eg, neighborhood deprivation index, social vulnerability index). However, studies have demonstrated high levels of correlation across all area-based SDOH indices.^49,50^ Fifth, only adults were included in this analysis of area-based exposures. Prior to the age of 18 years, these metrics of SDOH likely reflect parents’ social situation rather than that of the patients. Lastly, clinical risk factors of hypertension and obesity are also associated with both adverse SDOH and HCM outcomes. Therefore, adjusting for these covariates could underestimate the magnitude of the impact of SDOH on HCM. Since patients residing in lower income groups have greater disease severity by the traditional measures of wall thickness, this is likely contributing to a greater proportion of patients with adverse outcomes. However, adjusting for maximal LV wall thickness did not change the overall HRs or level of statistical significance (eTable in Supplement 1).

## Conclusions

In conclusion, per the results of this cohort study, there was a significant association between residing in an area with lower household income or higher SDI and adverse outcomes in patients with HCM. This analysis demonstrates that where someone lives may influence clinical outcomes, even in conditions with strong genetic etiologies like HCM, and one’s environment likely contributes to the heterogeneity of disease presentation. While area-based factors that are readily accessible in a clinical encounter may have utility to identify patients with HCM at higher risk of adverse outcomes, future studies are needed to identify solutions to reduce risk and improve access and care for patients with HCM who experience more adverse SDOH to improve the overall disease trajectory in these patients.

## Notes

Supplement 1. eTable. Adjusting for Left Ventricular Wall Thickness eFigure 1. Association of Household Median Income and SDI With Atrial Fibrillation in Patients With HCM eFigure 2. Relative Hazard of SDI and Income on Atrial Fibrillation, Stratified by Presence or Absence of a Pathogenic Sarcomere Variant

Supplement 2. Data Sharing Statement
