# Supplementary material for: Social Determinants of Health and Clinical Outcomes in Hypertrophic Cardiomyopathy
Source: JAMA Cardiol. 2026 Jan 7;11(2):165–74. doi: 10.1001/jamacardio.2025.4869 (PMC12780983; doi:10.1001/jamacardio.2025.4869)
Supplement: Supplement 1. — eTable. Adjusting for Left Ventricular Wall Thickness eFigure 1. Association of Household Median Income and SDI With Atrial Fibrillation in Patients With HCM eFigure 2. Relative Hazard of SDI and Income on Atrial Fibrillation, Stratified by Presence or Absence of a Pathogenic Sarcomere Variant [file jamacardiol-e254869-s001.pdf]

Supplemental Online Content

Hafeez N, Claggett BL, Owens AT, et al. Social determinants of health and clinical outcomes in hypertrophic cardiomyopathy. *JAMA Cardiol*. Published online January 7, 2026. doi:10.1001/jamacardio.2025.4869

eTable. Adjusting for Left Ventricular Wall Thickness
eFigure 1. Association of Household Median Income and SDI With Atrial Fibrillation in Patients With HCM
eFigure 2. Relative Hazard of SDI and Income on Atrial Fibrillation, Stratified by Presence or Absence of a Pathogenic Sarcomere Variant

This supplemental material has been provided by the authors to give readers additional information about their work.

© 2025 Hafeez N et al. *JAMA Cardiology*.

eTable. Adjusting for Left Ventricular Wall Thickness

A. Comparison of Lowest vs Highest Income Groups

	Adjusted for age at diagnosis, sex, HTN, BMI		Also adjusted for max LVWT	
**Composite**	**HR (95% CI)**	**p-value**	**HR (95% CI)**	**p-value**
**HF**	2.07 (1.77-2.42)	< 0.001	2.15 (1.82-2.53)	< 0.001
**VA**	1.31 (0.97-1.78)	0.08	1.29 (0.94-1.76)	0.116
**Overall**	1.52 (1.36-1.69)	< 0.001	1.51 (1.35-1.69)	< 0.001

B. Comparison of Most Deprived vs Least Deprived Groups

	Adjusted for age at diagnosis, sex, HTN, BMI		Also adjusted for max LVWT	
**Composite**	**HR (95% CI)**	**p-value**	**HR (95% CI)**	**p-value**
**HF**	1.48 (1.29-1.7)	< 0.001	1.51 (1.31-1.74)	< 0.001
**VA**	1.55 (1.15-2.09)	0.004	1.57 (1.16-2.15)	0.004
**Overall**	1.36 (1.22-1.50)	< 0.001	1.36 (1.23-1.51)	< 0.001

Cumulative incidence of events from age 18 for outcomes of interest with statistical significance determined by multivariate cox regression model correcting for age, sex, hypertension, and obesity (left panel) and corrected for age, sex, hypertension, obesity, and max left ventricular wall thickness (right panel). **A)** comparison of patients residing in lowest and highest income areas, **B)** comparison of patients residing in most and least deprived areas. Abbreviations: HTN, hypertension; LVWT, left ventricular wall thickness.

© 2025 Hafeez N et al. *JAMA Cardiology*.

eFigure 1. Association of Household Median Income and SDI With Atrial Fibrillation in Patients With HCM

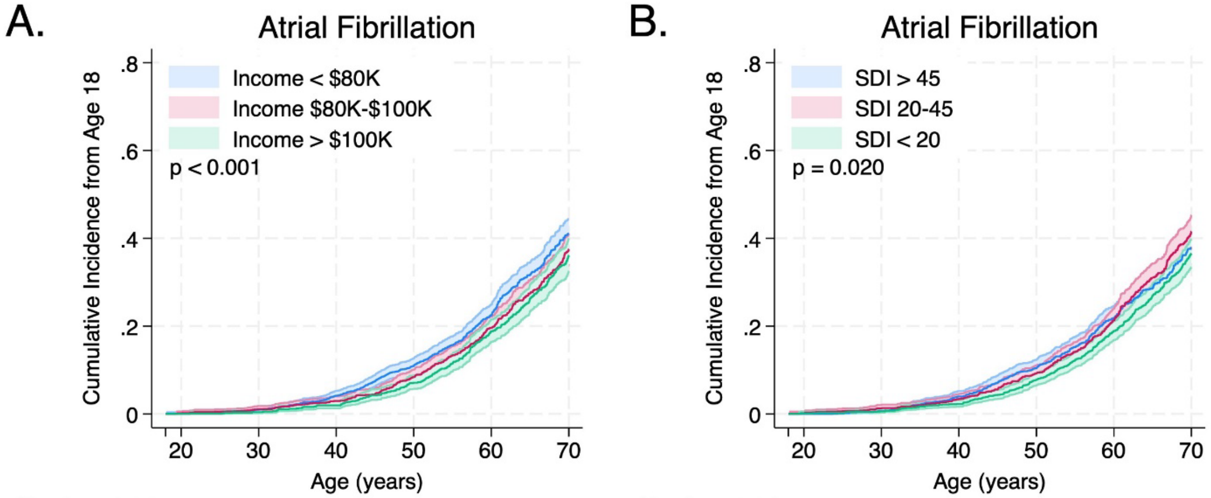

Number at risk

< $80K	1734	1660	1483	1197	772	304
$80-100K	1390	1329	1206	996	687	296
> $100K	1239	1199	1099	906	606	257

Number at risk

SDI > 45	1367	1302	1145	926	600	253
SDI 20-45	1358	1297	1166	949	640	273
SDI < 20	1638	1589	1477	1224	825	331

A) Cumulative incidence of events from age 18, stratified by household median income <$80,000, $80,000-$100,000, and >$100,000. **B)** Cumulative incidence of events from age 18 for outcomes of interest, stratified by SDI > 45, 20-45, and <20. Shaded areas indicate 95% CI. Statistical significance determined by multivariate cox regression model correcting for age, sex, hypertension, and obesity.

© 2025 Hafeez N et al. *JAMA Cardiology*.

eFigure 2. Relative Hazard of SDI and Income on Atrial Fibrillation, Stratified by Presence or Absence of a Pathogenic Sarcomere Variant

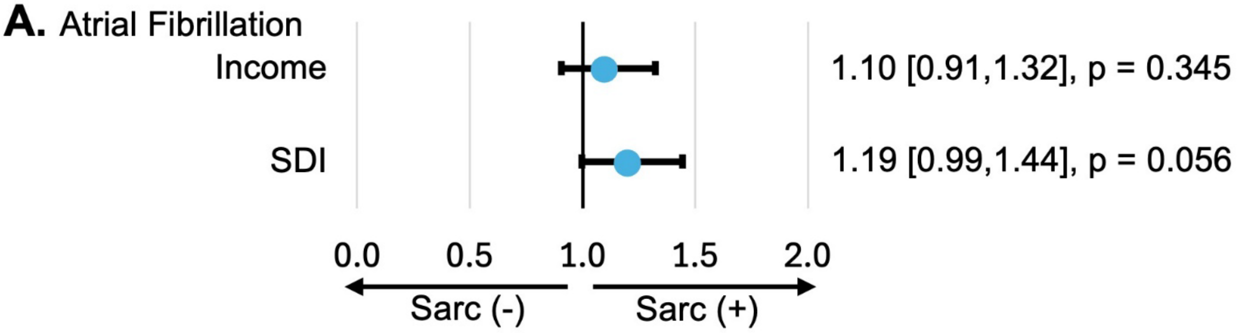

A) Forest plot demonstrating risk ratio of cumulative incidence of events from age 18 for atrial fibrillation, comparing hazard ratio of outcomes of sarcomere positive versus sarcomere negative patients. Error bars represent 95% confidence interval.

© 2025 Hafeez N et al. *JAMA Cardiology*.
