# Supplementary material for: Social Determinants of Health and Clinical Outcomes in Hypertrophic Cardiomyopathy
Source: JAMA Cardiol. 2026 Jan 7;11(2):165–74. doi: 10.1001/jamacardio.2025.4869 (PMC12780983; doi:10.1001/jamacardio.2025.4869)
Supplement: Supplement 2. — Data Sharing Statement [file jamacardiol-e254869-s002.pdf]

Data Sharing Statement

Hafeez. Social Determinants of Health and Clinical Outcomes in Hypertrophic Cardiomyopathy. *JAMA Cardiol*. Published January 07, 2026. doi:10.1001/jamacardio.2025.4869

Data
Data available: No

Additional Information
Explanation for why data not available: We are not able to provide individual level data from the SHaRe registry but can provide aggregate data on request.
